# Dynamic Volume Completion and Deformation

**DOI:** 10.1177/2041669517740368

**Published:** 2017-12-12

**Authors:** Peter Ulric Tse

**Affiliations:** Department of Psychological and Brain Sciences, 3728 Dartmouth College, Hanover, NH, USA

**Keywords:** 3D perception, binocular vision, contours/surfaces, depth, grouping, higher order motion, shape

## Abstract

A new class of dynamic volume completion is introduced, where image elements (e.g., occluding semi-ellipses placed at the edge of an object) can link across a gap between two or more objects, leading to the perception of illusory volumes that deform as those image elements are set into relative motion. These new demonstrations provide further evidence that volume completion is not dictated solely by contour relatability constraints, but is instead a dynamic process of 3D shape construction that also takes into account dynamic cues to object shape, even in the absence of any contour relatability whatsoever.

## Historical Background

Amodal completion of an object behind another occluding object, as well as modal completion of an object in front of its inducers, such as Kanizsa’s pacmen (e.g., Kanizsa, 1955, 1979; Kanizsa & Gerbino, 1982; Michotte, Thinés, & Crabbé, 1964), both result from rapid, constructive visual processes (Bruno, Bertamini, & Domini, 1997; De Wit, Bauer, Oostenveld, Fries, & Van Lier, 2006; De Wit & Van Lier, 2002; Gerbino & Salmaso, 1987; Vrins, De Wit, & Van Lier, 2009) that occur preattentively (Rauschenberger & Yantis, 2001; Rensink & Enns, 1998). The capacity to complete surfaces appears to begin within the first months of life (Kellman & Spelke, 1983; Kellman, Spelke, & Short, 1986), as is the case also for volume completion.

Initially, perceptual psychologists emphasized Gestalt organizational principles (Koffka, 1935), such as global stability, regularity, and simplicity of form to explain why image fragments complete the way that they do (e.g., Hochberg & McAlister, 1953), rather than other conceivable ways that would also be consistent with image cues, but which are rarely if ever perceived. Gestalt grouping laws are, however, rather unsatisfactory, and sometimes even circular explanations. One hears terms like *Praegnanz*, meaning essentially organizational simplicity, used to explain why one percept dominates. But when one tries to understand how an operation assessing such simplicity might be realized, at an algorithmic level, details are missing, or reference is made circularly to the percept itself. In general, the problem with Gestalt Psychology is that it asserts that parts group together because they follow grouping laws, without really explaining, at a mechanistic, neuronal or algorithmic level how such grouping operations take place. This lack of rigor alienated some perceptual scientists from the Gestalt approach. Gestalt Psychologists should be acknowledged for raising the problem of completion, but other approaches were needed to solve how this might be done by visual brain processes.

In search of rigor, the Gestalt approach was extended by a more concrete push toward conceiving of completion in terms of precise operations over the image. In particular, pairs of the global image cue of relative contour orientation were touted as the key inputs into modal and amodal completion operations (e.g., Kellman & Shipley, 1991; Wouterlood & Boselie, 1992). Kellman and Shipley (1991) formalized the Gestalt law of good continuation (Ullman, 1990; Wertheimer, 1923, 1938) and argued that two edges occluded by a single object would amodally or modally complete when the angle between their intersecting imaginary extensions (that have no reversals of curvature) subtended 90 degrees or more. This was an advance over Gestalt Psychology in that no appeal was made to abstract grouping laws left unexplained at an algorithmic (let alone a neural) level. Instead the goal was to reduce grouping operations to an algorithm that a computer vision system could carry out by extending and evaluating visible contours behind occluders.

This local-cue-driven account of completion of Kellman and Shipley (1991) or Wouterlood and Boselie (1992) was challenged by others who attempted to explain completion in terms of global regularities in the patterns of completing objects (e.g., Buffart, Leeuwenberg, & Restle, 1981; Sekuler, 1994; Van Lier, van der Helm, & Leeuwenberg, 1994, 1995).

A parallel line of thought, also derived from Gestalt Psychological grouping laws, attempted to understand amodal and modal completion in terms of surface completion on a common depth plane (Nakayama & Shimojo, 1992; Nakayama, Shimojo, & He, 1995; Nakayama, Shimojo, & Silverman, 1989). The key concept relating surface completion to amodal and modal completion is “border ownership” (see also Rubin, 1915). When there is a border in an image separating regions that project from two surfaces separated in depth, that border is projected from the nearer of those surfaces, namely, either from its edge or from its “rim,” which is the imaginary curve on a visible surface where the line of sight tangentially grazes that surface. Border-ownership occurs because, assuming no accidental or nongeneric alignment of surface edges, the border between two image regions can only project from one of those two projecting surfaces. The region that owns the border is taken to project from the occluding surface (i.e., that occludes the surface that projects to the region that does not own the border). The occluded surface can then continue behind the occluding surface and link with other occluded surfaces on the same depth plane because its corresponding image region is “unbounded” on the side where it does not own its border.

From all sides of this debate, regarding the mechanisms underlying perceptual completion, there was therefore great interest in describing the local image cues that allow the visual system to determine occlusion relationships. One image cue to occlusion includes contour tangent discontinuities, such as a T-junctions (Clowes, 1971; Huffman, 1971; Kellman & Shipley, 1991; Lowe, 1987; Malik, 1987; Nakayama et al., 1989; Waltz, 1975). Even though T-junctions are not necessarily present when occlusion is perceived (particularly in cases of surface interpenetration; see, e.g., Figure 22 in Tse & Albert, 1998), T-junctions are generically present when one surface occludes another surface that is separated from it in depth.

Thus, as of the mid-1990s, there were two dominant but related families of views regarding completion phenomena. The “good contour continuation view” was based on detecting local image cues to occlusion, such as T-junctions, and testing for good contour continuation over their nonvisible extensions, for example, behind an occluder. On this account, the inputs to the completion process are local junctions, contour tangent discontinuities, and contour orientations, whereas the outputs are global “units,” such as surfaces or holes. The appeal of this view is that these cues to occlusion are measurable in the image, so that given an image, a properly coded computer vision system could predict whether the visual system would complete disjoint fragments. Indeed, such a computer system could presumably carry out the same operations that underlie human visual completion, going some way toward fulfilling the dream of domain general computer vision, a prerequisite for any artificial intelligence system that might function in the world as we humans do.

In contrast, according to the “surface completion view,” the inputs into completion processes are image regions that do (or do not) own their border everywhere, and the outputs are surfaces whose edges and relative depths have been specified. The surface completion view involves completion over internal representations rather than image elements such as contours, because unbounded surfaces must be first inferred from image cues. They cannot be identified directly in the image as there are no surfaces or depths in a single image that would be detected at the retina or by the camera of a robot. Any computer vision system that could compute over surfaces would have to go well beyond image cues explicitly detectable in the image.

That said, these two families of views were not mutually exclusive. Several authors postulated interactions between contour interpolation and surface formation processes (e.g., Grossberg & Mingolla, 1985; Kellman & Shipley, 1991; Yin, Kellman, & Shipley, 1997).

In the late 1990s, several researchers began providing evidence (Albert & Tse, 2000; Tse, 1998, 1999a, 1999b, 2002; Tse & Albert, 1998; Van Lier, 1999; Van Lier & Wagemans, 1999) that these two “traditional” contour- and surface-based theories of completion were too limited, and instead developed an account of visual completion in terms of the linking of surfaces and the merging of the interpolated or constructed spatial (rather than material) insides that those surfaces enclose. On this third and more recent account, completion takes place at a higher volumetric level of representation, rather than at the level of contour or even surface completion. By “volume” is meant a 3D interpolated closed surface, including the invisible but sensed backside of a visible surface (Ekroll, Sayim, Van der Hallen, & Wagemans, 2016; Ekroll, Sayim, & Wagemans, 2013), and including, as well, the interpolated spatial inside that it encloses (Tse, 1999a, 1999b); again, no commitment is made to whether the completed volume is hollow or solid, or what material might fill the volume. The representation of a volume is presumed to be rooted in one of the shapes in 3D space; thus, for example, a solid or hollow ball, or one made of wood of rubber, would each evoke the same spherical volume percept, despite their substantial differences. Furthermore, two volumes are mergeable when their unbounded visible surfaces, rather than visible image contours, are connectable in 3D. This occurs when their visible portions can be extended into occluded space along the trajectories defined by their inferred surface curvatures, so that they merge into a common surface that has a backside, and the insides enclosed by those surfaces can completely merge.^1^

In the present short article, I offer demonstrations that volume completion is computed not only on the basis of static monocular cues but also on the basis of binocular depth cues among surfaces and contours placed at different depths. In addition, these demonstrations make apparent that volume completion takes into account dynamic aspects of the image sequence, such that volumes, once computed, can be updated to remain consistent with new relationships among image cues to 3D shape, even if that entails the construction of nonrigid illusory volumes that change their 3D shape over time. Previous authors have shown that nonrigid illusory surfaces can be perceived (e.g., Anderson, O’Vari, & Barth, 2011; Jain & Zaidi, 2011; Masuda et al., 2013; Masuda, Matsubara, Utsumi, & Wada, 2015; Weiss & Adelson, 2000). The present work goes farther by showing that nonrigid illusory volumes (i.e., closed surfaces) can also be perceived.

## Demonstrations

Many of these demonstrations (animated GIF files that can be played in Quicktime Player in Loop mode, or simply opened in a browser such as Firefox) take advantage of a fact, first described in Tse and Albert (1998), that there are cases of volumetric occlusion that do not give rise to image tangent discontinuities. For example, a cylindrical rod that penetrates some surface, such as water, will generically give rise to an elliptical contour in the image arising from those points where the rod meets the water. This ellipse will not exhibit T-, L-, or X-junctions in the image, and will generically lack image tangent discontinuities in the image (Tse & Albert, 1998). Taking advantage of this fact, placing an ellipse in the image can give rise to the perception that a cylindrical column is meeting or penetrating a surface. Building on this insight, I have placed ellipses near the edges of objects such as rectangles, to create the illusion that a cylindrical occluder meets the surface in question. What follows are various examples of illusory volumes that arise by exploiting this simple image-sequence construction strategy. What is particularly remarkable about these examples is that they can give rise to the illusion of rubbery 3D round (in cross-section) bands that link corresponding elliptical portions of the image. Note that the elliptical inducers are themselves rigid, but the completed volume that appears to link two such inducers can appear to be nonrigid, bending or bent, when, in the static instances of such inducer pairs, the completed volume would more typically not to be bent, and certainly not appear to be nonrigid.

In Online Movies 1(a) to (d) (both slow and fast, and white on black, and black on white variants), it appears that rubbery volumetric bands are attached to and link the balls as they change position randomly. These are discrete updates of position that result in apparently analog 3D shape changes of the rubbery bands. This suggests that volume completion facilitates the generation of illusory elastic bands. Note that even though the jumps are discrete in the image, the deformations in shape that the illusory bands appear to undergo appear relatively smoothly analog. This suggests that the interpolation of 3D shape changes may integrate data over a duration, rather than simply updating the inferred volumetric shape of the bands at each moment, independently of that inference made at other discrete moments. Future empirical work should work out what that temporal window of integration is.

The dynamic completion effect is diminished in the control case, shown in Online Movie 1(e), where the elliptical occluders that would be consistent with an occluding volume are replaced with rectangular occluders that are not consistent with volume completion; The rectangular occluders, unlike the elliptical occluders, are not consistent with elastic inducers in the world, so do not as readily lead to a percept of dynamic modal volume completion.

In Online Movies 2 to 4, positions are updated in an analog rather than discrete manner, and the 3D illusory volumes that are constructed to link inducer pairs appear to deform smoothly. Note that these movies are also consistent with another interpretation, which is one where objects with “elliptical bites” taken out of them are moving. Indeed, this interpretation is often the one people first see when given just a static frame from one of these movies. Under this interpretation, no illusory occluding, deforming volumes are perceived. This is ambiguity occurs because the elliptical portions of contour can either be “owned” (in the sense of border ownership) by the modally occluding volumetric bands, or by the objects with “bites” taken out of them. These image sequences are therefore visually bistable, like a Necker cube, and are likely subject to some degree of top-down control in flipping between these two interpretations, as occurs in the bar-cross-ellipse illusion (Caplovitz & Tse, 2006). But when given a dynamic version, most people spontaneously report seeing the deforming bands, and report, again spontaneously, that they appear to be made of something elastic, like rubber, taffy or dough. This suggests that the dynamic nature of the stimuli plays a role in the switch to an interpretation consistent with volume completion, just as sequentially occurring occlusion cues can facilitate amodal and modal surface completion in the nonvolumetric surface domain (McCarthy, Kohler, Tse, & Caplovitz, 2015).

Online Movies 5(a) to (d), both white and black versions, take advantage of binocular disparity to create the impression of volumetric deformation in depth. While it is possible to get some of the dynamic modal completion effect by looking at one of the images, the full 3D effect becomes more apparent upon crossed binocular fusion. The effect is most convincing when completion is allowed to take place somewhat in the visual periphery, as occurs when one smoothly pursues the top elliptical edge in Online Movies 5(a) and (b). In these cases, it can appear that there is a rubbery band connecting the two half ellipses (which in this case also have squares attached to them at the same depth, to better create the illusion of a cylindrical connection) deforms not only in one fronto-parallel plane, as in the above examples, but actually seems to deform by coming closer to and going farther away from the observer in depth. Thus, the volumetric representation of the rubbery cylindrical band linking the two “solid” cylindrical portions is updated in light of depth cues to create a shape that can deform in all three dimensions of space. In particular, when the object appears to jut forward, it appears to undergo an elongation or deformation in depth without breaking its connection with the other, more distant portion of the volumetric object. Moreover, when one sees the illusory elastic band linking the two visible portions, it appears to have illusory contours. Note that in the absence of binocular disparity, each individual monocular image would be unlikely to give rise to a percept of illusory volume completion, particularly when the “cylindrical” visible inducers are far from relatable in the image. Note that the rubbery band can appear to break at such points of poor image relatability. The point of illusory band breakage appears to differ among observers, and also to differ depending on the point of fixation. For example, some observers have reported that smoothly pursuing the top elliptical edge in Online Movies 5(a) and (b) leads to less frequent breakage than occurs when smoothly pursuing the bottom elliptical edge. Why this should be is not clear. But, in general, with binocular disparity, there is a range of image contour relationships where an illusory volumetric connector can smoothly and modally link the two inducers across depths, by bending in 3D space, rather like an elastic band or worm, even in cases that would fail to satisfy typical image contour relatability criteria.

The example in Online Movies 5(c) and (d) is actually bistable. Under one interpretation, the deforming volumetric connector can appear in front, when the inducers are taken to lie on the slanted supporting surfaces. But under another interpretation, those slanted surfaces become slanted windows through which one can see the deforming volumetric connector linked amodally behind the opaque vertical bar between the two windows. Under the former interpretation, illusory contours of the deforming volumetric connector can be seen, whereas under the latter interpretation, they are not seen, consistent with the idea that illusory contours follow specification of figure versus ground relationships, rather than dictate them (Kogo & Wagemans, 2013; Tse, 1999a).

Other examples of disparity-defined 3D curved surfaces exploit a phenomenon called “da Vinci stereopsis” (Cao & Grossberg, 2005; Nakayama & Shimojo, 1990; Wardle & Gillam, 2013). Leonardo da Vinci considered cases where an object occludes a more distant surface such that some portion of the more distant surface was occluded for one eye, but not the other. Online Movies 6(a) and (b) exploit a version of “reverse da Vinci stereopsis,” where one portion of an occluding surface or contour, rather than occluded surface, is visible to only one eye (see also Tse, in press). Online Movies 6(a) and (b) are constructed by moving two ellipses, one on either side of an “occluded rectangle.” Because reverse da Vinci Stereopsis involves a situation where an edge is only seen by one eye, a volume can be completed that modally completes the occluding contours visible to respective eyes into something like a single “slinky” that links the two half-ellipses into a single cylindrical volume. This illusory volume deforms as the visible elliptical edges move. Again, however, this example is perceptually bistable: One can either perceive the volumetric solution, in which case the volume appears to deform in shape and give rise to illusory contours, or one can perceive two independently moving ellipses, in which case no deforming volume or illusory contour is perceived. The same technique can give rise to a percept of translational apparent motion of a hockey puck-like volume, as in Online Movie 6(c), or transformational apparent motion (Tse, 2006; Tse, Cavanagh, & Nakayama, 1998; Tse & Logothetis, 2002) of a volume, as in Online Movie 6(d).

The preceding examples all involve dynamic modal volume completion. Online Movies 7(a) to (h) offer examples of dynamically deforming amodal volume completion. That is, under the interpretation of these image sequences, where a single object is take to move behind an occluding tube or column, the perceived volume appears to change its shape as it moves around the occluder. These demonstrations also make the point that the inferred volume is constructed over time, because at no time are there two visible portions of the moving snake or worm that could complete in a single image in the Online Movies 7(a) to (h) cases. In the examples shown in Online Movies 1 to 6, inducer pairs were present in the image. It could be argued that volume completion took place over static images which were then concatenated into a dynamic “movie” of a deformable volume. This is not a workable explanation for the Online Movie 7 cases. Here the perceived dynamically deforming volumes can only result from the construction of a deforming volume that links behind the occluder and over time, from one visible inducer at time t1 to a later one at time t2. Future work will have to work out the dynamics of temporal integration of volume completion over discrete image segments presented at different times, but it is likely to be comparable with the temporal dynamics found for spatiotemporal integration of nonclosed surfaces (McCarthy et al., 2015).

Online Movies 8(a) and (b) offer examples of apparent motion over discretely presented amodally completing volumes. In each case, a partially occluded worm occupies just three positions in sequence ABCB and so on. Under one interpretation, the worm in Online Movie 8(a) “slithers” upward around the pole. Under another, it flips upward and downward rather than slithering. Which of these two bistable interpretations is seen is subject to top-down control. In Online Movie 8(b), a portion of the worm is occluded by the pole, but is still taken to be present behind the pole. The duration that an occluded portion of a volume continues to be represented as being behind an occluder is an interesting focus for future research.

## Discussion

The goal of this article is to introduce examples of a new class of visual illusions where a modally or amodally completing volume is taken to deform its 3D shape over time. Most of these demonstrations take advantage of an image cue to volumetric occlusion that involves no image tangent discontinuities, first described in Tse and Albert (1998). When a cylinder penetrates or adheres to a 3D surface, it projects onto the image an elliptical boundary from the points on the surface or cylinder where the two surfaces meet or interpenetrate. This allows the strategic placement of an ellipse on an image to give rise to the impression of a 3D cylindrical occluder. Here two or more such elliptical occluders have been placed on separate surfaces, allowing a cylindrical volume to appear to link the two interpolated 3D occluders across a gap. This alone is remarkable, because it reveals the degree to which volumes are constructed on the basis of often sparse image cues. However, what is even more remarkable is the fact that moving these elliptical image cues, either within a depth plane, or between depth planes, results in the impression of a volume that deforms its shape in 3D to maintain the constructed connection between the two visible (elliptical) portions of the occluders. While past authors have investigated volume completion (Albert & Tse, 2000; Tse, 1998, 1999a, 1999b, 2002; Tse & Albert, 1998; Van Lier, 1999; Van Lier & Wagemans, 1999), and other authors have investigated dynamic illusory nonrigid open surfaces (e.g., Anderson et al., 2011; Jain & Zaidi, 2011; Masuda et al., 2013, 2015; Weiss & Adelson, 2000), the present work is the first to explore the intersection of these domains. This is the case, extensively demonstrated here, of dynamic illusory nonrigid closed surfaces or volumes.

## Note

1 It is not surprising that we evolved to represent the invisible sides of visible surfaces; if a siamang swinging through a tree only grabbed the visible sides of branches, it would surely lose its grip and fall. To be able to swing from a branch, it must grab the invisible far side of the top of the branch. Indeed, much of what we call seeing involves perceiving patterns that are literally invisible, whether regarding the far sides of objects, causation, or other minds. The unconscious inference of such patterns, on the basis of what is visible in the image sequence, poses a deep problem for computer vision algorithms which, at least currently, lack a complex world model, including of other minds, that affords the construction and seeing of the invisible.
